# The GLP-1R Agonist Exendin-4 Attenuates Hyperglycemia-Induced Chemoresistance in Human Endometrial Cancer Cells Through ROS-Mediated Mitochondrial Pathway

**DOI:** 10.3389/fonc.2021.793530

**Published:** 2021-12-20

**Authors:** Yu Zhang, Juan Cheng, Jing Li, Junxian He, Xiaomao Li, Fen Xu

**Affiliations:** ^1^Department of Gynecology, Third Affiliated Hospital, Sun-Yet Sen University, Guangzhou, China; ^2^Department of Endocrinology and Metabolism, The Third Affiliated Hospital of Sun Yat-sen University, Guangzhou, China; ^3^Guangdong Provincial Key Laboratory of Diabetology, Guangzhou, China

**Keywords:** chemoresistance, cisplatin, cytotoxicity, Exendin-4, endometrial cancer

## Abstract

This study aimed to assess the effects of the antidiabetic drug Exendin-4 (Exe-4), a GLP-1 receptor agonist, on the response of human endometrial cancer cells to chemotherapy under high glucose (HG) conditions. Cell viability was detected using a cell counting kit (CCK)-8. Cell apoptosis and reactive oxygen species (ROS) levels were measured by flow cytometry. Gene expression was evaluated by real-time PCR and immunoblotting. The chemotherapeutic drug cisplatin (DDP) dose-dependently inhibited both human endometrial adenocarcinoma Ishikawa and HEC1B cells, a response reversed by HG. Meanwhile, Exe-4 attenuated hyperglycemia’s effect by elevating intracellular lactate dehydrogenase (LDH) and ROS production. Similarly, DDP-induced elevation of intracellular rhodamine123 was attenuated by HG, and Exe-4 reversed HG’s impact. The chemoresistance genes multidrug resistance-associated protein 1 (MRP1) and P-glycoprotein (Pgp) were upregulated. At the same time, topoisomerase II (TOPO II) was downregulated under HG conditions, suggesting HG-induced chemoresistance. Exe-4 did not significantly influence the above genes. DDP downregulated Bcl-2 and Bcl-XL and upregulated Bax, cytosolic cytochrome c, and PARP under normal glucose (NG) versus HG conditions, and Exe-4 attenuated these effects. Upstream of Bax/Bcl, acetylated P53 was upregulated by DDP and downregulated by HG, whose effect was reversed by Exe-4. DPP treatment significantly induced apoptosis and cell cycle arrest in the S phase under NG, and HG reduced these effects. Prolonged exposure to HG induces DDP chemoresistance in human endometrial cancer cells but is alleviated by Exe-4.

## Introduction

Endometrial cancer is the sixth most common neoplasm in women worldwide. All other malignancies have been shown to improve survival rates since the 1970s; endometrial and cervical cancers have not followed this trend (1). Indeed, the incidence of endometrial cancer has increased over time, with the highest rates being observed in high-income countries. These countries are such as North America and Europe, in addition to Asia, which shows the largest increase (2). Epidemiological studies have shown that the risk factors for endometrial cancer include early menarche, late menopause, nulliparity, hormone replacement therapy, obesity, and diabetes (2). The prevalence of diabetes is increasing worldwide, with 12.8% of adults affected in 2016 (3). There will be at least 629 million people living with diabetes by 2045 without proper intervention (4). Hyperglycemia is associated with diabetes and obesity, which in turn have strong associations with cancer development (1, 5, 6), progression (7), and mortality (8). That is particularly the case for breast, colorectal, pancreas, endometrium, and urinary tract cancers (9).

Chemotherapy is an important therapeutic method in cancer, and multidrug resistance (MDR) represents the key factor determining treatment failure, leading to tumor progression or recurrence (10). The sustained hyperglycemia is associated with a reduced cancer response rate to chemotherapy or radiotherapy (11–13). However, the underlying causes of drug resistance in malignancy are multifactorial. For example, chemoresistance to cisplatin involves increased platinum-DNA adduct elimination due to nucleotide excision repair (NER) (14). Diabetes profoundly alters energy metabolism. Both insulin deficiency and insulin resistance are characterized by inefficient mitochondrial coupling and excessive reactive oxygen species (ROS) production. This irreversibly causes DNA damage and alters cellular functions (15). In addition, increased ROS in cancer cells contributes to the biochemical and molecular changes necessary for tumor initiation, promotion and progression, and tumor resistance to chemotherapy (16).

Therefore, hyperglycemia is also considered a factor involved in chemotherapy resistance (17). Indeed, a recent study showed that high glucose concentrations reduce mitochondrial DNA damage, Bax/Bcl-2 and Bax/Bcl-XL ratios, and sub-G1 phase cells associated with antitumor drug-induced cytotoxicity (13). One of the mechanisms of MDR is the overexpression of ATP binding cassettes (ABC) transporters such as P-glycoprotein (Pgp) and other MDR-related proteins (18–21). Meanwhile, chemotherapeutic reversing the first hurdle of redox-controlled cellular uptake channels (Pgp, MDR2, and MDR3) trigger concerted effects of phase I/II metabolic enzymes with a temporal redox-regulated axis (22).

Interestingly, plumbagin, a molecule well-known for anti-diabetes properties in animal models (23). It enhances cisplatin’s anticancer efficacy by increasing intracellular ROS in human tongue squamous cell carcinoma (24). Still, whether hypoglycemic agents could attenuate high glucose (HG)-induced impaired chemotherapy efficacy remains largely unknown. Therefore, this study aimed to assess the effects of the anti-diabetes drug Exendin-4 (Exe-4), a GLP-1 receptor agonist (25), on the response of human endometrial cancer cells to chemotherapy after prolonged exposure to HG. This study investigated the two most widely used human endometrial adenocarcinoma cell lines- Ishikawa and HEC1B. This was because that both are now recognized based on the difference in etiopathology, clinical characteristics, and genetics (26). The findings of this study provide some insights into the mechanisms by which hypoglycemic drugs such as GLP-1 receptor agonists could suppress chemoresistance.

## Materials and Methods

### Cell Culture and Modeling

Two human endometrial adenocarcinoma cell lines- Ishikawa (high differentiation) and HEC1B (low differentiation) were obtained from the American Type Culture Collection (ATCC, Rockville, MD, USA). They were grown as a sub-confluent monolayer in RPMI-1640 base medium (GIBCO, Invitrogen Inc., Carlsbad, CA, USA) containing 2 mM L-glutamine (KeyGen Biotech Co., Beijing, China), 1% (v/v) antibiotic/antimycotic solution (KeyGen Biotech), and 10% (v/v) fetal bovine serum (FBS) (GIBCO), at 37°C in a humid environment with 5% CO_2_.

All cells were cultured for up to 8 weeks in a medium containing normal glucose (NG, 5.56 mM) or HG (25 mM) (27) with mannitol (Absin Bioscience Inc., China; M, 25 mM) for osmotic control. Preliminary experiments showed that the use of mannitol did not influence the cells. The glucose concentrations of 5.56 mM and 25 mM in this study correspond to glycemia in fasting healthy individuals and hyperglycemia in diabetic patients with very poorly controlled sugar levels.

### Cell Viability

Cells were grown under NG or HG for eight weeks and treated for 48 h with DDP (Luoxin Pharmaceutical Group, Beijing, China) at various concentrations (serial dilutions from 400.00 μM to 1.56 μM, and 0 μM) and/or Exe-4 (Sigma, St Louis, MI, USA). A preliminary study assessed Exe-4 at 1, 10, and 100 nM. No significant inhibitory effects were found at 10 nM on Ishikawa and HEC1B cells under HG or NG conditions for eight weeks (**Supplementary Table 1**). In order to assess viability, the cells were stained for 2 h at 37°C in a culture medium containing CCK-8 solution (Biosharp, Beijing, China), and absorbance was read at 450 nm. The viability of untreated cells was considered 100%, and the results were expressed as a percentage of viable cells in each experimental condition versus untreated cells. The inhibitory concentration 50 (IC50), defined as the concentration of the drug decreasing cell viability by 50%, was calculated with the CompuSyn software.

### Lactate Dehydrogenase (LDH) Release

For LDH release measurement, the cells were grown under NG or HG conditions for eight weeks. After further incubation for 48 h with Exe-4 (10 nM) and/or DDP (25 µM), the supernatants were collected and centrifuged at 12,000 ×g for 30 min. LDH activity (in U/L) was measured in the cleared supernatants with an LDH kit (KeyGEN BioTECH) to confirm the cytotoxic effects of chemotherapeutic agents by reading absorbance at 450 nm (37°C).

### Measurement of Cellular ROS Production

For eight weeks, cells were grown under NG or HG conditions and incubated for 48 h with Exe-4 (10 nM) and/or DDP (25 µM). After washing with PBS, the cells were collected and resuspended at 1×10^6^/ml, incubated with DCFH-DA (KeyGEN BioTECH; 10 μM) for 20 min at 37°C. Then, the cells were washed with a serum-free cell culture medium three times. Cellular ROS levels were detected by flow cytometry on a Becton-Dickinson FACS Calibur (BD Biosciences, Franklin Lake, NJ, USA).

### Detection of Apoptosis

For eight weeks, cells were grown under NG or HG conditions and incubated for 48 h with Exe-4 (10 nM) and/or DDP (25 µM). After digestion with 0.25% pancreatin without EDTA and two PBS washes, 5×10^5^ cells were resuspended with 500 μL of binding buffer and mixed with 5 μL of Annexin V-FITC (KeyGEN BioTECH) and 5 μL of propidium iodide at room temperature away from light for 10 min. The samples were analyzed for apoptosis by flow cytometry on a Becton-Dickinson FACS Calibur.

### Pgp and MRP Activity Assessment

The efflux of rhodamine 123, a substrate of Pgp and MRP, was measured as an index of Pgp plus MRP activity. The cells were grown under NG or HG conditions for 8 weeks and incubated for 48 h with Exe-4 (10 nM) and/or DDP (25 µM). After PBS washing and detachment with a cell dissociation solution, the cells were resuspended at 1×10^6^ cells/mL in 1 mL of DMEM medium containing 5% FBS. The samples were maintained at 37°C for 20 min in the presence of 10 µg/mL rhodamine 123 (KeyGEN BioTECH). After one PBS wash, the cells were resuspended in 500 µL of PBS, and intracellular rhodamine content, which is inversely related to its efflux. Then, cells were analyzed using flow cytometry on a Becton-Dickinson FACS Calibur, with excitation and emission wavelengths of 488-505 nm and 515-575 nm, respectively.

### Quantitative Real-Time Polymerase Chain Reaction (qRT-PCR)

Cells were grown under NG or HG conditions for 8 weeks, incubated for 48 h with Exe-4 (10 nM) and/or DDP (25 µM), and washed with PBS. Total RNA was extracted with the TRIzol reagent (Invitrogen Inc., Carlsbad, CA, USA). RNA concentration and purity were detected on a NanoDrop as OD260/280 of 1.8-2.1. According to the manufacturer’s instructions, total RNA (2 μg) was reverse transcribed into cDNA in a final reaction volume of 20 μl with the cDNA first-strand synthesis kit (RR036B, Takara Bio, Otsu, Japan). The qRT-PCR primers (**Supplementary Table 2**) were designed with Primer 6. Quantitative PCR was carried out in a final volume of 20 µL with One Step TB Green™ PrimeScript™ RT-PCR Kit II (SYBR Green) (RR086B, TaKaRa). PCR amplification was performed at 95°C for 30 s, followed by 40 cycles of 95°C for 30 s and 60°C for 1 min on a StepOne Real-Time PCR (System Thermo Fisher Scientific, USA). Data were analyzed by the 2^-ΔΔCt^ method, with β-actin used for normalization (28–30).

### Western Blot

Cells were grown under NG or HG conditions for eight weeks, incubated for 48 h with Exe-4 (10 nM) and/or DDP (25 µM), washed twice with PBS, and lysed for 1 h with ice-cold lysis buffer (50 mM Tris–HCl, 150 mM NaCl, 5 mM EDTA, pH 7.4). They were supplemented with the protease inhibitor cocktail set III in Total Protein Extraction Kit (KGP250, Kaiji Biotechnology, China), 1 mM sodium orthovanadate, 1 mM phenylmethanesulfonylfluoride, 1 mg/ml aprotinin, 50 mM sodium fluoride, and 1% Triton X-100. Cell lysates were centrifuged for 15 min at 14,000 rpm at 4°C. Proteins were subjected to SDS-PAGE and subsequently transferred onto PVDF membranes. The blots were blocked with 5% non-fat milk in PBS at room temperature for 1 h and incubated overnight with various primary antibodies (**Supplementary Table 3**). The membranes were washed with 0.1% v/v PBS-Tween and subjected to 1 h of incubation with sheep anti-rabbit IgG-HRP (KGAA35, Kaiji Biotechnology, China) or Sheep anti-mouse IgG-HRP (KGAA37, Kaiji Biotechnology, China) secondary antibodies (1:5000) in 5% w/v PBS-Tween. Detection and quantitation were performed by Imaging on a G: BOX chemiXR5, and the data were analyzed with the Gel-Pro32 software.

### Cell Cycle Analysis

The cells were grown under NG or HG conditions for eight weeks and incubated for 48 h with Exe-4 (10 nM) and/or DDP (25 µM). They were digested with 0.25% pancreatin (KeyGEN BioTECH) without EDTA and washed with PBS twice. The single-cell suspension was fixed with 70% ethanol overnight at 4°C, washed with PBS, and incubated with 100 μL of RNase A (KeyGEN BioTECH) at 37°C for 30 min. Next, 400 μL of PI (KeyGEN BioTECH) was added and incubated away from light at 4°C for 30 min. Afterward, flow cytometry detection was done at 488 nm excitation wavelength (Becton-Dickinson FACS Calibur, USA).

### Statistical Analysis

Data are presented as means ± standard error of the mean (SEM) and were checked for normal distribution. Data were analyzed by one-way analysis of variance (ANOVA) followed by Tukey’s test. P < 0.05 was considered statically significant. SPSS 22.0 (IBM, Armonk, NY, USA) was used for data analysis.

## Results

### Hyperglycemia May Help Alleviate Cancer Cells From the DDP-Induced Cytotoxicity, an Effect Attenuated by Exe-4

**Figure 1:**
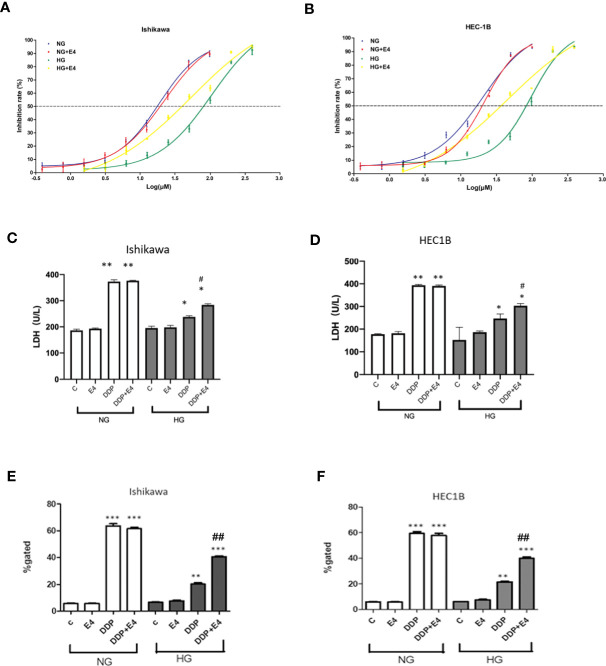
Hyperglycemia may help alleviate cancer cells from the DDP-induced cytotoxicity, an effect attenuated by Exe-4. Ishikawa **(A)** and HEC1B **(B)** were grown under NG and HG conditions for eight weeks, respectively, then further incubated for 48 h with or without Exe-4 (10 nM) to assess cell viability. IC50 was calculated as the concentration of DDP that kills 50% (blue line) of cells. The IC50 values of DDP for Ishikawa in the NG, NG + Exe-4, HG, and HG + Exe-4 groups were 16.7 3µM, 18.569 μM, 75.283 μM, and 36.92 μM, respectively **(A)**. Those of DDP for HEC1B in the NG, NG + Exe-4, HG and HG + Exe-4 groups were 15.428 μM, 18.621 μM, 74.857 μM, and 35.774 μM, respectively **(B)**. **(C, D)** LDH levels in supernatants from Ishikawa **(C)** and HEC1B **(D)** cells cultured under HG versus NG with various treatments. E and **(F)** ROS amounts in Ishikawa **(E)** and HEC1B **(F)** cells cultured under HG or NG with different treatments. ^∗^p < 0.05, ^**^p < 0.01, ^***^p < 0.001 versus control group or corresponding treatment group under NG; ^#^p < 0.05, ^##^p < 0.01 versus DDP group under HG.

The IC50 values of DDP in cells treated with HG were elevated compared with those obtained under NG conditions in both Ishikawa and HEC1B cells (**Figures 1A, B**). In addition, under NG conditions, Exe-4 did not considerably alter the IC50 values of DDP in both cell lines. However, the IC50 values of DDP were reduced in both cell lines after Exe-4 administration. This indicates that the cells were sensitized by Exe-4 to DDP after HG-induced resistance (**Figures 1A, B**). Specifically, the IC50 values of 16.726 μM and 18.569 μM were obtained for DDP in Ishikawa cells of the NG and NG + Exe-4 groups, respectively; 75.283 μM and 36.920 μM were determined, respectively, for the HG and HG + Exe-4 groups. In HEC-1B cells, the IC50 values were 15.428 μM and 18.621 μM for DDP in the NG and NG + Exe-4 groups, respectively; 74.857 μM and 35.774 μM were determined for the HG and HG + Exe-4 groups, respectively. These findings indicated that Exe-4 reversed hyperglycemia-induced DDP cytotoxicity impairment in Ishikawa and HEC1B cells.

Next, LDH release was assessed as an index of cytotoxicity. As shown in **Figures 1C, D**, LDH levels in supernatants were significantly lower in Ishikawa and HEC1B cells, respectively, cultured under HG versus NG at the same DDP dose and incubation time. In addition, Exe-4 increased LDH release in both cell lines under HG conditions. These data confirmed that Exe-4 attenuated hyperglycemia-induced chemoresistance to DDP in Ishikawa and HEC1B cells.

Intracellular ROS levels were examined in Ishikawa and HEC1B cells. The results showed that ROS amounts were reduced in the HG groups of both cell lines than the NG groups at the same DDP dose and incubation time (**Figures 1E, F**). Interestingly, treatment with Exe-4 resulted in increased ROS levels in both cell lines under HG conditions. They further demonstrated that Exe-4 alleviated hyperglycemia-associated DDP cytotoxicity impairment in Ishikawa and HEC1B cells (**Figures 1E, F**).

**Figure 2:**
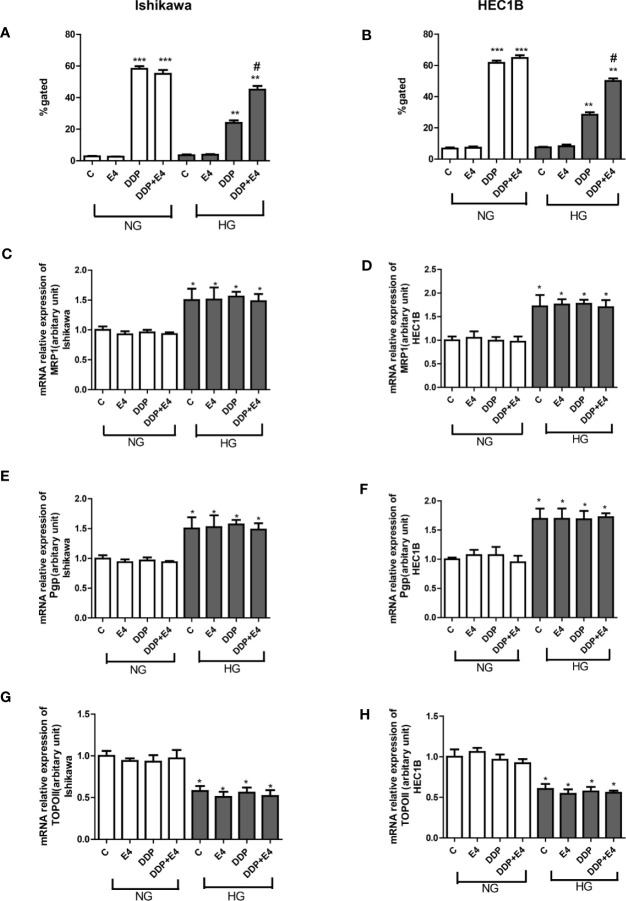
Effects of Exe-4 on rhodamine 123 amounts and the expression levels of MRP1, Pgp, and TOPO II genes after treatment with DDP under NG and HG conditions. **(A, B)** Cells were cultured with rhodamine 123 to assess Pgp and MRP activity flow-cytometrically. Measurements were performed in triplicate. ^∗∗∗^p < 0.001 DDP (25 µM), DDP (25 µM) + Exe-4 (10 nM) *vs.* control group and Exe-4 (10 nM) in Ishikawa **(A)**/HEC1B cells **(B)** cultured in NG; ^∗∗^p < 0.01 DDP (25µM), DDP (25µM) + Exe-4 (10 nM) *vs.* control group and Exe-4 (10 nM) in Ishikawa/HEC1B cells cultured in HG; ^*^p < 0.05 DDP (25 µM) + Exe-4 (10 nM) *vs.* DDP (25 µM) in Ishikawa/HEC1B cells cultured in HG. ^#^p < 0.05 DDP (25 µM) + Exe-4 (10 nM) *vs.* DDP (25 µM) under HG conditions. At the same experimental conditions, cells were analyzed by quantitative real-time polymerase chain reaction (RT-qPCR) for MRP1, Pgp, and TOPO II genes in Ishikawa **(C, E, G)** respectively) and HEC1B **(D, F, H)**, respectively) cells. Measurements were performed in triplicate, and data are mean ± SEM; ^∗^p < 0.05 Ishikawa and HEC1B cells cultured in HG *vs.* cultured in NG.

To confirm that the changes of extracellular LDH and intracellular ROS levels induced by mitochondrial membrane potential change, intracellular rhodamine 123 amounts (as an index of Pgp and MRP activity) in Ishikawa and HEC1B cells were examined. The results showed that intracellular rhodamine 123 amounts were significantly higher in the DDP groups compared with the control groups, and HG attenuated this effect; however, Exe-4 reversed the effect of HG (**Figures 2A, B**). qRT-PCR showed that MRP1 and Pgp gene expression levels were increased. In contrast, topoisomerase II (TOPO II) gene expression was decreased by HG (**Figures 2C** **–H**), and Exe-4 did not significantly influence the above genes. MRP5 and MRP8 levels showed no significant changes among the various treatment groups in both cell lines (**Supplementary Figure 1**).

### Exe-4 Attenuates Hyperglycemia-Induced Decrease of the Apoptotic Effects of DDP in Ishikawa and HEC1B Cells

**Figure 3:**
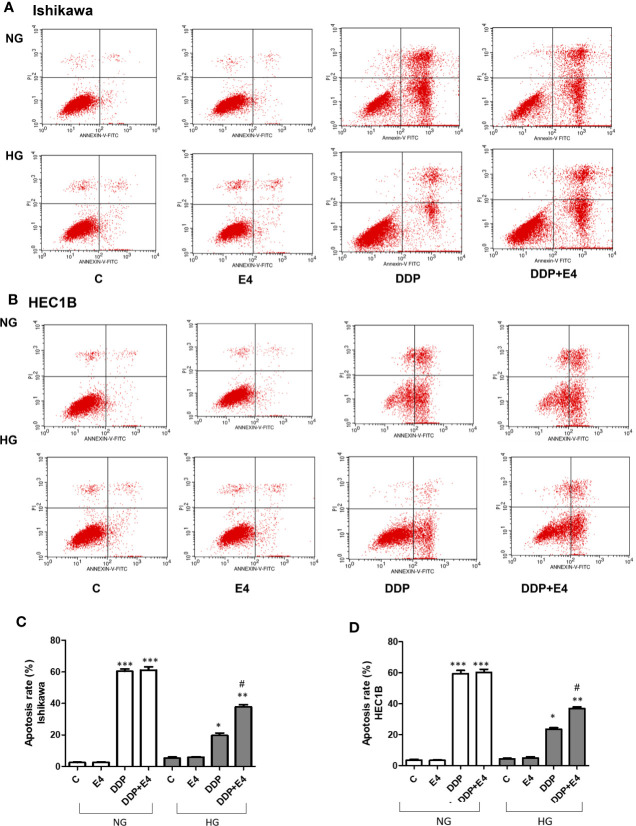
Effects of Exe-4 in high glucose (HG) on cell apoptosis in the absence or presence of cisplatin (DDP) in human endometrial cancer (Ishikawa and HEC1B) cells. Cells were incubated for 48 h with Exe-4 (10 nM), DDP (25 µM), DDP (25 µM) + Exe-4 (10 nM), respectively, and apoptosis was detected by Annexin-V FITC/PI double staining. Measurements were performed in triplicate. ^∗∗∗^p < 0.001 DDP (25ROS formed in triplicate TC/PI double staining cancer (Ishikawa and HEC1B) cells. ^∗^p < 0.05 DDP (25 µM), ^∗∗^p < 0.01 DDP (25µM) + Exe-4 (10 nM) *vs.* control group and Exe-4 (10 nM) in Ishikawa/HEC1B cells cultured in HG; ^#^p < 0.05 DDP (25 µM) + Exe-4 (10 nM) *vs.* DDP (25 µM) in Ishikawa/HEC1B cells cultured in HG. Flow cytograms are presented in Ishikawa **(A)** and HEC1B **(B)** cells, quantitated in **(C, D)**, respectively.

Next, apoptosis was assessed by Annexin-V FITC/PI double-staining in both Ishikawa and HEC1B cells. As shown in **Figure 3**, hyperglycemia abolished the pro-apoptotic effects of DDP, and Exe-4 attenuated the effect of HG in both cell lines (**Figure 3**). Next, effectors involved in apoptosis were evaluated.

### Exe-4 Reverses Hyperglycemia-Induced Reduction of DDP’s Effects on the Cell Cycle in Ishikawa and HEC1B Cells

**Figure 4:**
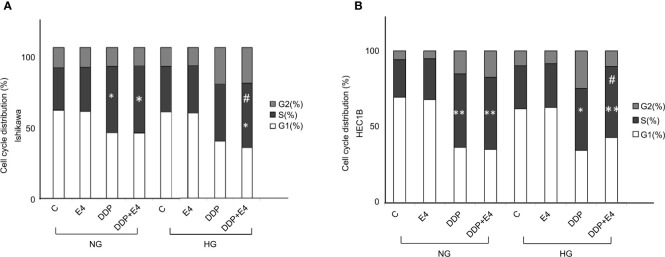
Effects of Exe-4 and DDP under high glucose (HG) on cell cycle in Ishikawa and HEC1B cells. Cells were cultured for 8 weeks under NG and HG conditions, incubated for 48 h with Exe-4 (10 nM), DDP (25 µM), and DDP (25 µM) + Exe-4 (10 nM), respectively, and analyzed for DNA content by FACS analysis. The panels represent the distribution of cells in different phases of the cell cycle. Measurements (n = 3) were performed. ^∗^p < 0.05 DDP (25 µM), DDP (25 µM) + Exe-4 (10 nM) *vs.* control group and Exe-4 (10 nM) in Ishikawa cells cultured in NG; ^∗^p < 0.05 DDP (25µM) + Exe-4 (10 nM) *vs.* Exe-4 (10 nM) in Ishikawa cells cultured in HG; ^#^p < 0.05 DDP (25µM) + Exe-4 (10 nM) *vs.* DDP (25 µM) in Ishikawa cells cultured in HG **(A)**. ^∗^p < 0.01 DDP (25 µM), DDP (25 µM) + Exe-4 (10 nM) *vs.* control group and Exe-4 (10 nM) in HEC1B cells cultured in NG; ^∗^p < 0.05 DDP (25 µM), ^**^p < 0.01 DDP (25 µM) + Exe-4 (10 nM) *vs.* control group and Exe-4 (10 nM) in HEC1B cells cultured in HG; ^#^p < 0.05 DDP (25µM) + Exe-4 (10 nM) *vs.* DDP (25µM) in HEC1B cells cultured in HG **(B)**.

Further, cell cycle distribution was assessed under NG and HG conditions in both cell lines. As shown in **Figure 4**, cell cycle arrest in the S phase was more pronounced in the NG groups than in the HG groups in both cell lines. Meanwhile, Exe-4 reversed the effect of HG, increasing the amounts of cells in the S phase after DDP treatment under HG conditions (**Figure 4**).

### Molecular Mechanisms of Exe-4 in Reversing HG-Induced Chemotherapy Resistance in Ishikawa and HEC1B Cells

**Figure 5:**
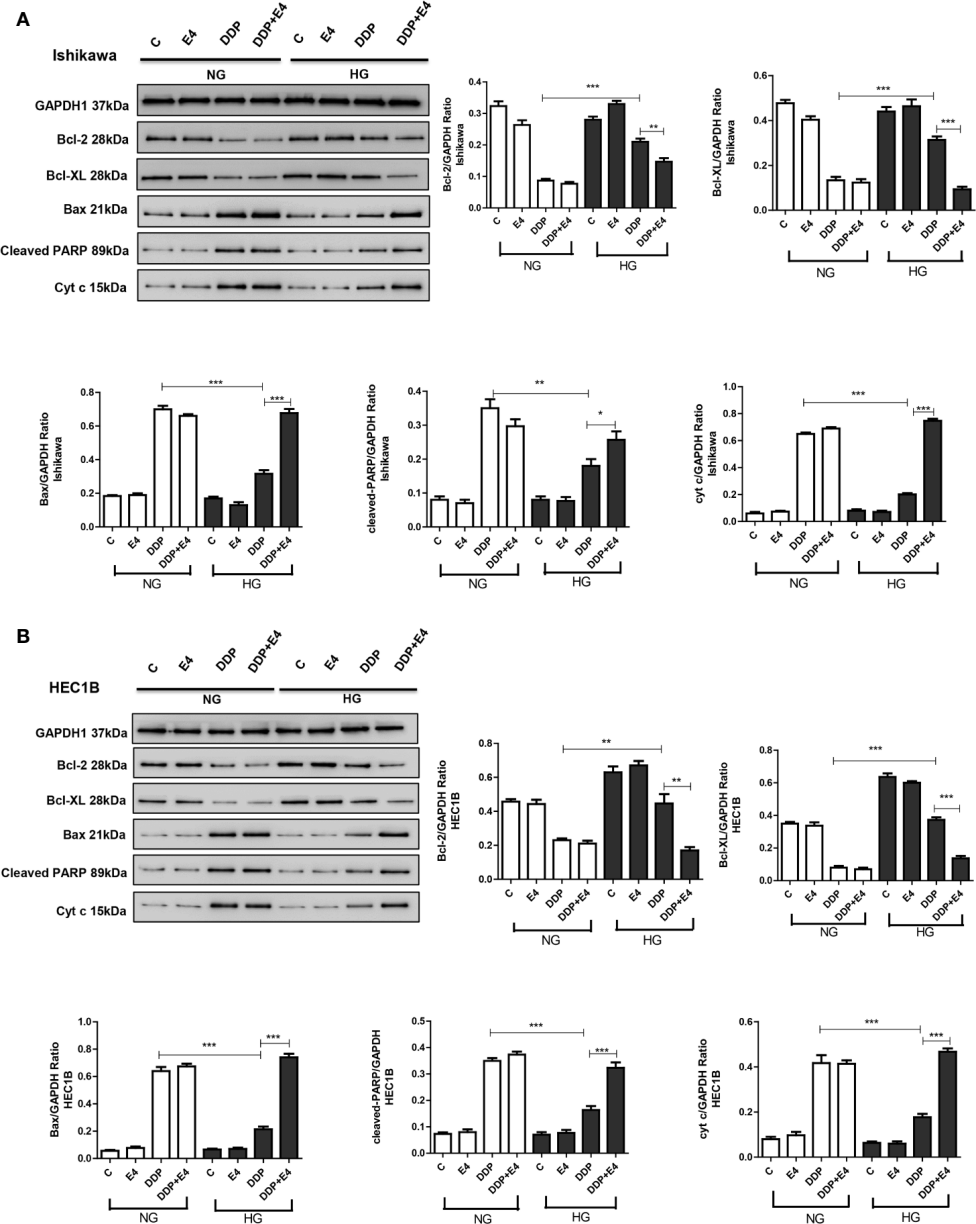
Effects of high glucose (HG), DDP, and Exe-4 on Bcl-2, Bcl-XL, Bax, cleaved PARP, and cytosolic cytochrome c levels in Ishikawa and HEC1B cells. Protein levels were assessed by immunoblot, with GAPDH as a loading control. Data for Ishikawa **(A)** and HEC1B **(B)** cells are shown. Measurements were performed in triplicate. *p < 0.05, **p < 0.01, ***p < 0.001, DDP in HG *vs.* DDP in NG, DDP+Exe-4 *vs.* DDP in HG.

As shown in **Figures 5A, B**, DDP downregulated Bcl-2 and Bcl-XL more pronounced under normal glycemia than hyperglycemia in Ishikawa and HEC1B cells. Bcl-2 and Bcl-XL were upregulated in the DDP + Exe-4 + HG group compared with the DDP + HG group. Moreover, DDP significantly upregulated Bax under HG and NG conditions, but less in the HG groups than in the NG groups. Meanwhile, Exe-4 reversed the above effects of HG in both cell lines (**Figure 5**). Furthermore, cytochrome c (cyt c) amounts in the cytosol were markedly decreased after DDP treatment in Ishikawa and HEC1B cells cultured under HG versus NG conditions. Exe-4 significantly reduced the effect of HG to increase cytosolic cyt c levels under HG conditions. This corroborates the above data obtained for Bax, a known mediator of the release of multiple factors, including cytosolic cyt c, that trigger apoptosis. We next assessed Poly (ADP-ribose) polymerase (PARP) as a caspase activation index and an enzyme involved in DNA repair. As shown in **Figure 5**, cleaved PARP decreased after DDP treatment more under HG conditions than the NG groups in both cell lines. Meanwhile, Exe-4 reversed the effect of HG on cleaved PARP amounts after DDP treatment.

**Figure 6:**
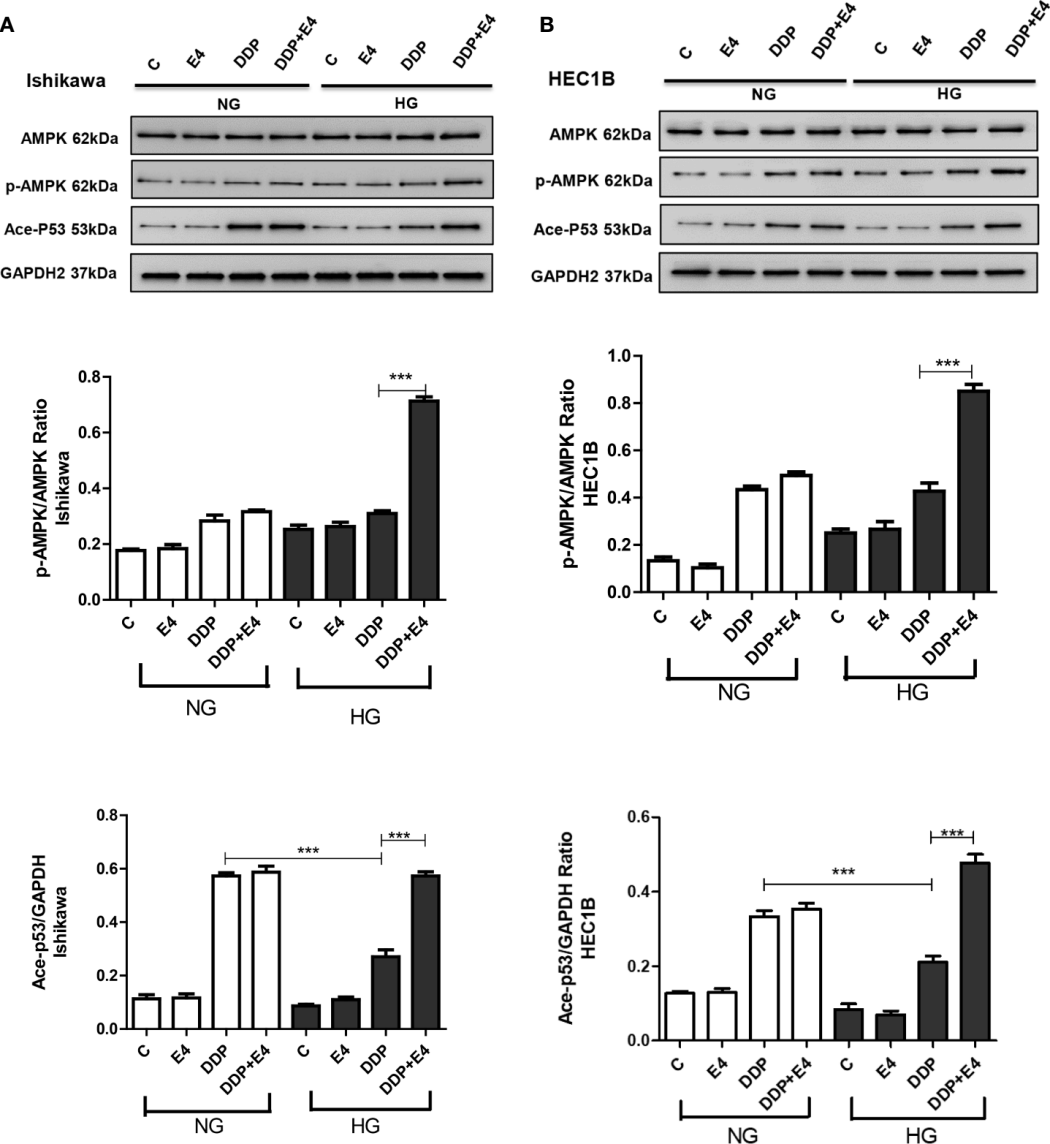
Effects of high glucose (HG), DDP, and Exe-4 on AMPK, p-AMPK, and acetylated P53 levels in Ishikawa and HEC1B cells. Protein levels were assessed by immunoblot, with GAPDH as a loading control. Data for Ishikawa **(A)** and HEC1B **(B)** cells are shown. Measurements were performed in triplicate. ***p < 0.001, DDP in HG *vs.* DDP in NG, DDP+Exe-4 *vs.* DDP in HG.

The role of AMPK has been proven as the key to a series of complex molecular events, including apoptosis (31). In this study, the p-AMPK/AMPK ratio and acetylated p53 amounts were assessed in both cell lines in the contexts of HG and NG. As shown in **Figures 6A, B**, p-AMPK/AMPK ratios and acetylated p53 amounts increased in the HG + DDP + Exe-4 groups compared with the HG + DDP groups in both groups Ishikawa and HEC1B cell lines. These findings indicated that Exe-4 reversed the effects of HG in these cell lines *via* AMPK signaling, regulating the ROS-mediated mitochondrial pathway of apoptosis.

## Discussion

DDP is the most popular and effective anticancer drug for female reproductive tract malignancies (32). Several cancers, including endometrial cancer, showed higher mortality rates in patients with hyperglycemia (33), which caused resistance to chemotherapy (34–37). Indeed, Yang et al. (34) showed that hyperglycemia was associated with oxaliplatin resistance in colorectal cancer cells. Bergamino et al. (35) showed that high fasting glucose levels were independently associated with non-small cell lung cancer outcomes. Ikemura et al. (36) showed in mice that hyperglycemia-induced resistance oxaliplatin and 5-fluorouracil.

Recently, it has been reported that hyperglycemia promotes chemoresistance by decreasing mitochondrial DNA damage, Bax/Bcl-2 and Bax/Bcl-XL ratios, and sub-G1 phase cells associated with antitumor drug-induced cytotoxicity in human colon adenocarcinoma cells (13). That is supported by Garufi et al. (37), who showed that hyperglycemia reduced p53 apoptotic activity, reducing cell response to cancer drugs. In the present study, cell viability was significantly lower in Ishikawa and HEC1B cells cultured under HG or NG conditions after treatment with DDP. However, hyperglycemia protected the cancer cells from the DDP-induced cytotoxicity. It is known that chemotherapeutic agents, including DDP, cause mitochondrial respiratory chain ROS generation, which plays a significant role in the inhibition of cancer progression (38).

Further, based on the previous reports by Bergandi et al. (13) and Garufi et al. (37), hyperglycemia has effects on the ROS-mediated mitochondrial pathway, pro-apoptotic (Bax), and anti-apoptotic (Bcl-2 and Bcl-XL) proteins. These are involved in the regulation of the cell cycle, DNA repair, and replication. The Bcl-2 protein is known to inhibit apoptosis by regulating the mitochondrial membrane potential and cytochrome c release needed for activation of caspase-9. PARP, used as a caspase activation index and as an enzyme involved in DNA repair. Besides, the AMPK role has been proven as the key to a series of complex molecular events, including apoptosis. P53 regulates cell cycles and cell apoptosis, and acetylated P53 is associated with stress. Therefore, Bcl-2, Bcl-XL, Bax, cleaved PARP, AMPK, p-AMPK, cytosolic cytochrome c, and acetylated P53 levels in Ishikawa and HEC1B cells were evaluated by immunoblot to reveal the effects of HG, DDP, and Exe-4. DDP exerted important cytotoxic effects on tumor cells in this study, as reflected by increased LDH release and ROS production. These were used as indexes of cytotoxicity, and Exe-4 attenuated hyperglycemia-induced lowering LDH release and ROS amounts in Ishikawa and HEC1B cells treated with DDP. The level changes of extracellular LDH and intracellular ROS result from mitochondrial membrane potential changes (39), which are reflected by rhodamine 123 amounts. As shown above, intracellular amounts of rhodamine 123 (also considered an index of Pgp plus MRP activity) were significantly increased after treatment with DDP, whose effects were attenuated by HG.

Currently, little is known about the effects of hypoglycemic drugs on the response of cancer to chemotherapy. Therefore, this study focused on the effect of the widely used antidiabetic peptide Exe-4, a GLP-1 receptor agonist, on DDP chemotherapy in endometrial cancer in the context of hyperglycemia-associated chemoresistance, exploring the underlying mechanism. Exe-4 attenuated the protective effect of hyperglycemia. Studies have revealed that Exe-4 could inhibit cancer cell growth *via* different mechanisms (40–43). Krause et al. (40) showed that exenatide induces cancer cell autophagy and prevents chemoresistance through mTOR modulation. Kanda et al. (41) showed that liraglutide prevented Ishikawa cell growth by enhancing autophagy. In the study by Zhang et al. (42), the growth of Ishikawa cells was inhibited by Exe-4, and the results suggest that AMPK is involved in the process. Similar results were reported by Nomiyama et al. (43) in prostate cancer, in which Exe-4 decreased cell proliferation through ERK-MAPK signaling. In this study, we preliminarily assessed Exe-4 cytotoxicity. Dose-dependent inhibition of endometrial cancer cells was observed, with 10 nM Exe-4 showing no significant inhibitory effects on Ishikawa and HEC1B cells for 8 weeks under both HG and NG conditions. In agreement, MRP1 and Pgp gene expression levels were increased while TOPO II gene expression was decreased under HG conditions. Yang et al. (34) showed that hyperglycemia was associated with oxaliplatin resistance in colorectal cancer cells, which could be reversed by metformin, supporting the present study. Still, Exe-4 did not significantly regulate the expression of the above genes, which deserves further investigation.

To summarize the findings obtained from both the human endometrial adenocarcinoma cell lines- Ishikawa and HEC1B cells, there is no significant difference among these two cell lines. Nonetheless, we only used *in vitro* assay; therefore, insulin secretion and/or glucose-lowering effects of Exe-4 cannot be expected in this case.

This study has limitations. (i) Only a small panel of genes was investigated, which cannot determine the exact mechanisms being involved in the role of the Exe-4. (ii) This study is highly descriptive, and mechanisms need to be investigated further. (iii) In this study, *in vitro* assay was employed. Thus, whether Exe-4 shows the potential additional efficacy glucose-dependently was not explored. (iv) Moreover, in this study, the combination of Exe-4, cisplatin, and high glucose medium showed the efficacy, and mechanism of glucose-dependent and cisplatin-dependent efficacy of Exe-4 need to be explored *in vivo* in the future.

## Conclusions

These data provide some insights into the molecular mechanisms involved in chemoresistance induced by hyperglycemia and demonstrate that hypoglycemic drug, GLP-1 receptor agonist-Exe-4, could help alleviate such chemoresistance in endometrial cancer.

## Data Availability Statement

The original contributions presented in the study are included in the article/**Supplementary Material**. Further inquiries can be directed to the corresponding authors.

## Author Contributions

Conceptualization: YZ, XL, and FX. Data curation: JC. Formal analysis: JL and JH. Funding acquisition: YZ, JC, XL, and FX. Investigation: YZ, JC, and JL. Methodology: JC, JL, and JH. Resources: JH. Software: JH. Supervision: XL and FX. Validation: XL and FX. Visualization: XL and FX. Roles/Writing - original draft: YZ, JC, and JL. Writing - review and editing: YZ and FX. All authors contributed to the article and approved the submitted version.

## Funding

This study was supported by the National Natural Science Foundation of China Grant [grant number 81970741 and 81670782 to FX]; The Local Innovative and Research Teams Projects of Guangdong Pearl River Talents Program [grant number 2017BT01S131 to FX)]; The Science and Technology Planning Project of Guangdong Province of China [grant number 2017A020215020 to YZ]; and the Medical Science and Technology Research Fund of Guangdong Province of China [grant number A2018293 to JC].

## Conflict of Interest

The authors declare that the research was conducted in the absence of any commercial or financial relationships that could be construed as a potential conflict of interest.

## Publisher’s Note

All claims expressed in this article are solely those of the authors and do not necessarily represent those of their affiliated organizations, or those of the publisher, the editors and the reviewers. Any product that may be evaluated in this article, or claim that may be made by its manufacturer, is not guaranteed or endorsed by the publisher.

## Supplementary Material

Supplementary Figure 1 Effects of high glucose (HG), DDP, and Exe-4 on MRP5 **(A, C)** and MRP8 **(B, D)** levels in Ishikawa and HEC1B cells.
