# Supplementary figures and images for: The GLP-1R Agonist Exendin-4 Attenuates Hyperglycemia-Induced Chemoresistance in Human Endometrial Cancer Cells Through ROS-Mediated Mitochondrial Pathway
Source: Front Oncol. 2021 Dec 20;11:793530. doi: 10.3389/fonc.2021.793530 (PMC8721044; doi:10.3389/fonc.2021.793530)

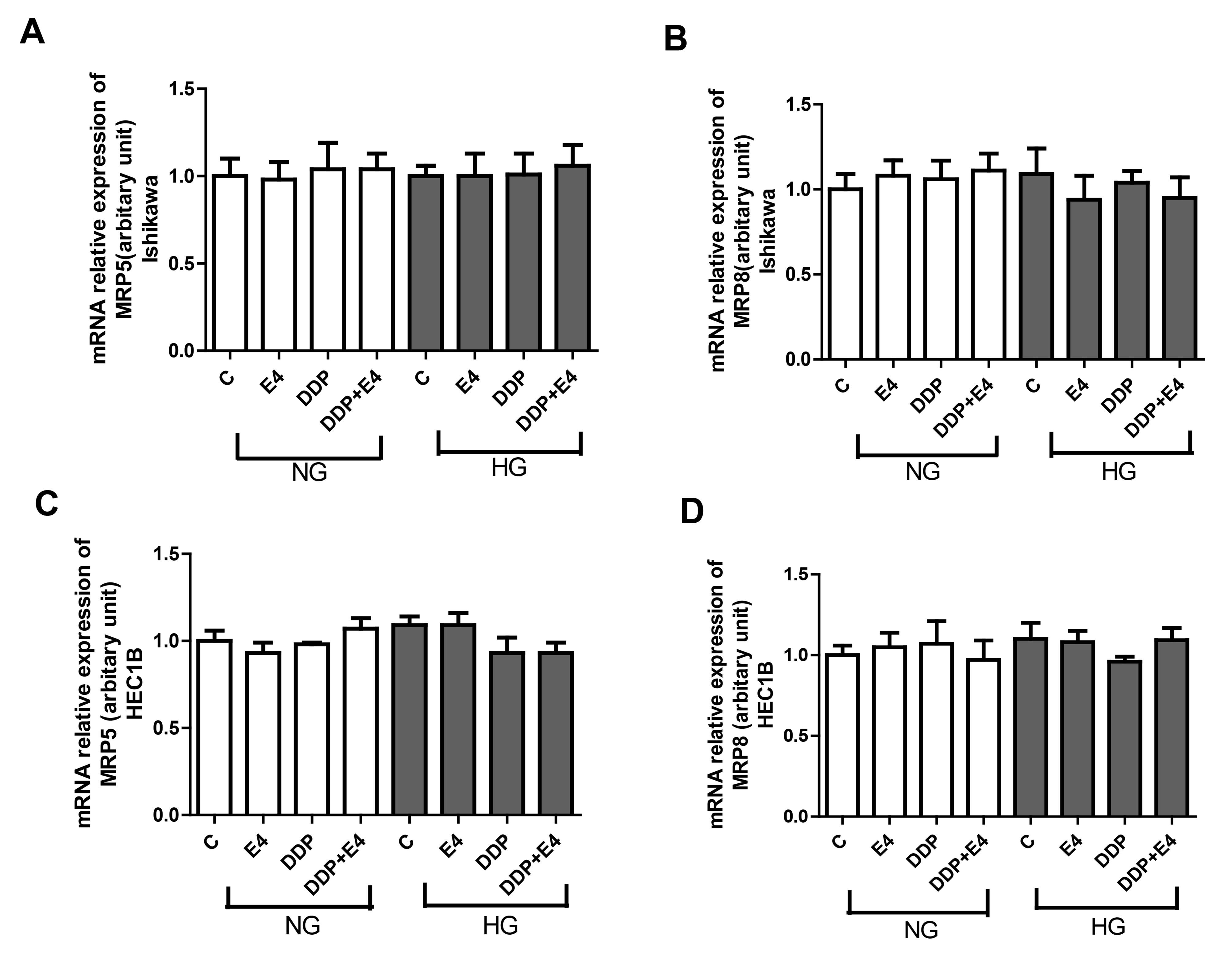

Supplement: Supplementary Figure 1 — Effects of high glucose (HG), DDP, and Exe-4 on MRP5 (A, C) and MRP8 (B, D) levels in Ishikawa and HEC1B cells. [file Image_1.tif]
