# Supplementary material for: The GLP-1R Agonist Exendin-4 Attenuates Hyperglycemia-Induced Chemoresistance in Human Endometrial Cancer Cells Through ROS-Mediated Mitochondrial Pathway
Source: Front Oncol. 2021 Dec 20;11:793530. doi: 10.3389/fonc.2021.793530 (PMC8721044; doi:10.3389/fonc.2021.793530)
Supplement: Supplementary file 2 [file Table_1.docx]

Supplementary Table S1 Inhibitory effects of Exendin-4 on Ishikawa and HEC1B cells under HG or NG conditions for 8 weeks

|  | Concentration | Ishikawa | Ishikawa (HG) | HEC-1B | HEC-1B (HG) |
| --- | --- | --- | --- | --- | --- |
| Exendin-4 | 100nM | -0.29% | 17.48%^**^ | 4.45%^*^ | 15.56%^**^ |
|  | 10nM | 0.67% | 3.36% | 1.65% | 3.82% |
|  | 1nM | 1.63% | 2.80% | -0.74% | 1.27% |

*, p<0.05 vs 0nM; **, p<0.01 vs 0nM
