# Supplementary material for: The GLP-1R Agonist Exendin-4 Attenuates Hyperglycemia-Induced Chemoresistance in Human Endometrial Cancer Cells Through ROS-Mediated Mitochondrial Pathway
Source: Front Oncol. 2021 Dec 20;11:793530. doi: 10.3389/fonc.2021.793530 (PMC8721044; doi:10.3389/fonc.2021.793530)
Supplement: Supplementary file 3 [file Table_2.docx]

Supplementary Table S2 Primers used for qRT-PCR

| Gene | Forward primer (5’-3’) | Reverse primer (5’-3’) |
| --- | --- | --- |
| *MRP1* | TGGACTTCGTTCTCAGGCACAT | CTCCTTCGGCAGACTCGTTGAT |
| *MRP5* | TCCTGTCCATCGTGTGCCTGAT | TGACCGCAGAATACAGCCATCC |
| *MRP8* | GCCTATCTCGTGCCATGTCCTC | GGTTGTCATCCTCCTCGCTCTC |
| *Pgp* | CATTCCTCCTGGAAATTCAACCT | CTTCAAGATCCATTCCGACCTC |
| *TOPO II alpha* | TGGAAACAGCCAGTAGAG | ATCTTTGTCCAGGCTTTG |
| *β-actin* | CCTGGGCATGGAGTCCTGTG | TCTTCATTGTGCTGGGTGCC |

*MRP1, ATP-binding cassette, sub-family C (CFTR/MRP) member 1 (ABCC1); MRP5, ATP-binding cassette, sub-family C (CFTR/MRP) member 5 (ABCC5); MRP8, ATP-binding cassette, sub-family C (CFTR/MRP) member 8 (ABCC8); Pgp, ATP-binding cassette, sub-family B (MDR/TAP) member 1 (ABCB1); TOPO II alpha, topoisomerase II alpha; β-actin, beta actin*.
