# Supplementary material for: The GLP-1R Agonist Exendin-4 Attenuates Hyperglycemia-Induced Chemoresistance in Human Endometrial Cancer Cells Through ROS-Mediated Mitochondrial Pathway
Source: Front Oncol. 2021 Dec 20;11:793530. doi: 10.3389/fonc.2021.793530 (PMC8721044; doi:10.3389/fonc.2021.793530)
Supplement: Supplementary file 4 [file Table_3.docx]

Supplementary Table S3 Antibodies used for immunoblot

| Primary antibody | | Dilution | Manufacturer | Catalog number | % gel for detection | |
| --- | --- | --- | --- | --- | --- | --- |
| Rabbit anti-GAPDH | | 1:10000 | Kaiji Biotechnology, China | KGAA002 | 10% | |
| Mouse anti-Bcl-2 | | 1:200 | Santa Cruz Biotechnology, USA | 7382 | 12% | |
| Mouse anti-bcl-xl | | 1:200 | Santa Cruz Biotechnology, USA | 8392 | 12% | |
| Mouse anti-Bax | | 1:200 | Santa Cruz Biotechnology, USA | 7480 | 12% | |
| Rabbit anti-PARP | | 1:1000 | Cell Signaling Technology , USA | 9532 | 10% | |
| Rabbit anti-Cyto-c | | 1:1000 | Cell Signaling Technology , USA | 11940 | 15% | |
| Rabbit anti-AMPK | | 1:1000 | Cell Signaling Technology , USA | 2352 | 10% | |
| Rabbit anti-P-AMPK | | 1:1000 | Cell Signaling Technology , USA | 2335 | 10% | |
| Rabbit anti-ace-p53 | | 1:1000 | Abcam, UK | 183544 | 12% | |
|  | |  |  |  |  | |
|  | | | | |  |  |
